# Keratinocytes derived from chicken embryonic stem cells support Marek’s disease virus infection: a highly differentiated cell model to study viral replication and morphogenesis

**DOI:** 10.1186/s12985-015-0458-2

**Published:** 2016-01-07

**Authors:** Mathilde Couteaudier, Katia Courvoisier, Laetitia Trapp-Fragnet, Caroline Denesvre, Jean-François Vautherot

**Affiliations:** INRA — Université François-Rabelais de Tours, UMR 1282 Infectiologie et Santé Publique, ISP, F-37380 Nouzilly, France

**Keywords:** Marek’s disease virus, Chicken keratinocytes, Viral replication, Viral morphogenesis

## Abstract

**Background:**

Marek’s disease is a virus disease with worldwide distribution that causes major losses to poultry production. Vaccines against Marek’s disease virus, an oncogenic alphaherpesvirus, reduce tumour formation but have no effect on virus shedding. Successful horizontal virus transmission is linked to the active viral replication in feather follicle epithelial cells of infected chickens, from which infectious viral particles are shed into the environment. The feather follicle epithelium is the sole tissue in which those infectious particles are produced and no in vitro cell-systems can support this highly efficient morphogenesis. We previously characterized embryonic stem-cell-derived keratinocytes, showing they display a marker-gene profile similar to skin keratinocytes, and therefore we tested their susceptibility to Marek’s disease virus infection.

**Findings:**

We show herein that keratinocytes derived from chicken embryonic stem-cells are fully permissive to the replication of either non-pathogenic or pathogenic Marek’s disease viruses. All viruses replicated on all three keratinocyte lines and kinetics of viral production as well as viral loads were similar to those obtained on primary cells. Morphogenesis studies were conducted on infected keratinocytes and on corneocytes, showing that all types of capsids/virions were present inside the cells, but extracellular viruses were absent.

**Conclusions:**

The keratinocyte lines are the first epithelial cell-line showing ectodermal specific markers supporting Marek’s disease virus replication. In this in vitro model the replication lead to the production of cell-associated viral progeny. Further work will be devoted to the study of relationship between 3D differentiation of keratinocytes and Marek’s disease virus replication.

## Findings

Keratinocytes of the feather follicle epithelium (FFE) are major target cells of the oncogenic alphaherpesvirus *Gallid herpesvirus type 2* (GaHV-2), or Marek’s disease virus (MDV) [1, 2]. Only these cells produce mature enveloped viral particles and thus form the only known source of environmental dissemination [2–4]. Using primary avian cells to replicate MDV in vitro [5, 6], the viral morphogenesis leads to a low number of these mature enveloped particles [7]. Recently, we derived the first chicken keratinocytes clones from chicken embryonic stem cells (K-cESCs) [8]. In the current report, we investigated whether these differentiated cell-lines are permissive to MDV infection and whether MDV morphogenesis is comparable to FFE.

**Fig. 1:**
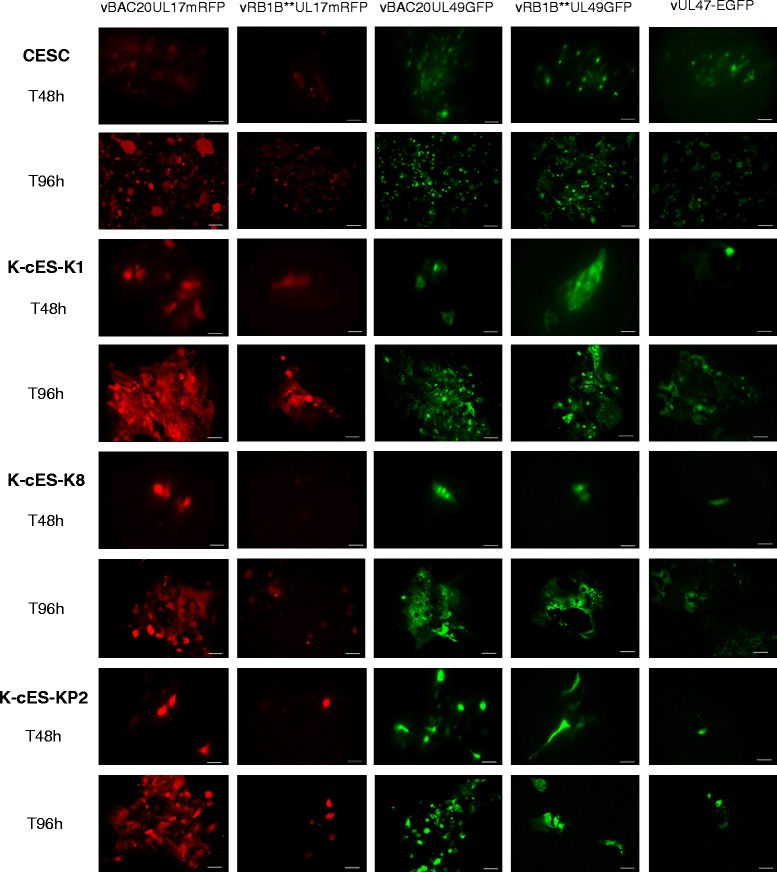
Chicken keratinocytes, K-cESCs, support the replication of recombinant fluorescent MDVs tagged on different tegument protein. CESCs and K-cESCs (−K1, −KP2 or -K8) were infected by co-culture with sorted-cells infected with either vBAC20UL17mRFP, or vRB1B**UL17mRFP, or vBAC20UL49GFP, or vRB1B**UL49GFP, or vUL47-EGFP and observed at 48 h and 96 h post-infection. Scale bars represent 50 μm

To examine the permissiveness to MDV infection, K-cESCs or primary chicken embryonic skin cells (CESCs), our standard cell culture system for MDV, were co-cultivated with sorted CESCs [9], infected by one of the following recombinant fluorescent MDV, vBAC20UL17mRFP [10], vRB1B**UL17mRFP (same construct as in 10 in the virulent RB1B backbone – C. Denesvre, personal communication), vBAC20UL49GFP [9], vRB1B**UL49GFP [11] and vUL47-EGFP [12]. For each cell-type, the level of infection was estimated from the development of plaques and the enlargement of one viral plaque, monitored over the time (Fig. 1). In both assays, we recorded increases in the levels of infection for the 5 viruses and in all cell-types, at similar levels in K-cESCs and in CESCs. Therefore, K-cESCs supported the replication of attenuated (vBAC20) as well as very virulent MDV (vRB1B), regardless of the tagged proteins (pUL17, pUL47 or pUL49).

**Fig. 2:**
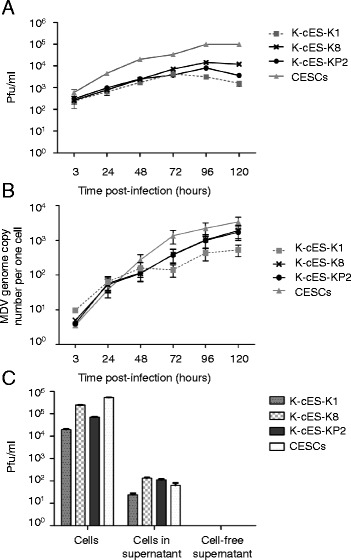
MDV replication in K-cESCs. Kinetics of infection of the vBAC20UL17mRFP on K-cESCs and CESCs, determined every 24 h from 3 to 120 h. Titres determined from two wells are given either in pfu/ml (**a**) or in MDV genome copy number/cell by qRT-PCR (**b**). Bars represent the mean ± SEM of viral loads. **c** Infectivity of the vBAC20UL17mRFP from K-cESCs or CESCs cells floating in the supernatant and from cell free supernatants. Statistical analyses were performed by Kruskal-Wallis one-way ANOVA test followed by two-tailed Mann–Whitney test. Analyses were done by using the software GraphPad Prism 5. No statistical significant difference between K-cESCs and CESCs was observed

To further explore the replication of MDV in K-cESCs, kinetics of infection of vBAC20UL17mRFP were studied on K-cESCs and CESCs. Five × 10^5^ cells were co-seeded with 7000 mRFP-sorted vBAC20UL17mRFP infected cells, in six-well culture plates in duplicate. Every 24 h, cells in two wells were harvested, and the virus was titrated by quantification of plaque forming units (pfu) [13] and of genome copy number per cell by qPCR [11, 14]. The kinetics of vBAC20UL17mRFP replication on K-cESCs and CESCs were similar, reaching a plateau at 96 h with a slightly lower viral production on K-cESCs than on CESCs (Fig. 2a). In all cells the MDV genome copy number increased rapidly, reaching almost the same level in K-cES-K8 and-KP2 than in CESCs (Fig. 2b). No significant differences in infectious titres or genome copy number could be observed between CESCs and K-cESCs (Kruskal-Wallis one-way ANOVA test followed by two-tailed Mann–Whitney test). From this initial experiment, we concluded that MDV replicates efficiently in K-cESCs.

K-cESCs in culture, as primary keratinocytes [15], undergo their terminal differentiation to form corneocytes that will naturally exfoliate in the supernatant [8]. We investigated whether infectious particles were associated with the corneocytes from cultures at day 5 post-infection by performing viral infectious titrations using either floating cells harvested in the supernatant or cell-free supernatants clarified by two centrifugations at 3000 × g. Significant viral titres were associated with floating cells from all cell-types including CESCs (Fig. 2c). In contrast, no viral infectivity could be detected in the clarified cell-free supernatants (Fig. 2c), indicating the absence of cell-free infectious particles in the supernatants of K-cESCs and CESCs, even when infected cells were submitted to sonication (60 s with a Vibra-cell™ 75455 ultrasonic vibrator at an intensity setting of 40 using a CV26 probe) before centrifugation and filtration of supernatants (data not shown).

**Fig. 3:**
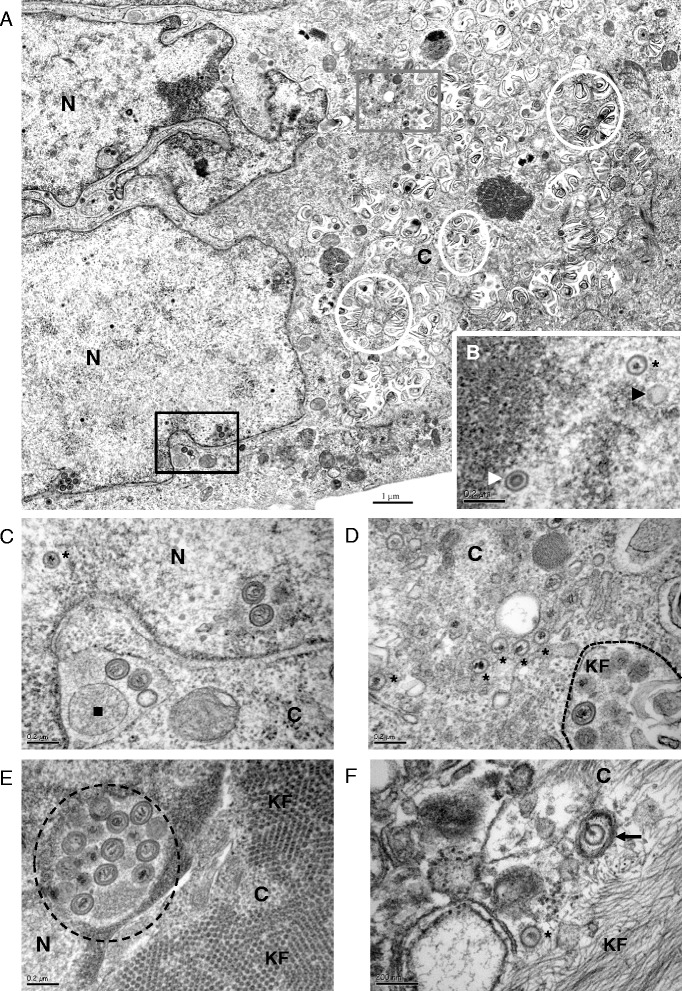
Viral morphogenesis in K-cESCs infected with vBAC20UL17mRFP. **a** K-cES-KP2 infected cell. **b** Enlargement of a part of the nucleus of K-cES-KP2 infected cell showing typical A, B and C capsids. **c** Enlargement of the lower boxed area on picture A showing cisternae of the nuclear membrane containing 2 primary enveloped viral particles and a granular material embedded in a single membrane (black square) Primary enveloped particles in nuclear membrane cisternae are often accompanied by single membrane embedded material, as if the primary envelopment of viral capsids was concomitant to the embedding of cellular material; 2 primary enveloped particles in a nuclear vacuole are present on the right. **d** Enlargement of the area boxed in grey in the cytoplasm of K-cES-KP2 cell on picture A. Numerous C capsids in the cytoplasm are close to a cytoplasmic vacuole containing primary enveloped particles. **e** Another K-cES-KP2 infected cell showing at least 11 viral particles in a cisternae close to the nuclear membrane (black circle). Note the abundant network of transversally sectioned fibres in the cytoplasm (KF). **f** Enveloped mature particle in a K-cES-K1 infected cell (black arrow). N : Nucleus; C : Cytoplasm; Black Stars : C capsids; Black triangle : A capsids; White triangle : B capsids; Black dashed circle : primary enveloped particles; White circle : degenerating lamellar bodies; KF : Keratin filaments

Viral morphogenesis was studied by transmission electron microscopy (TEM) [16] in keratinocytes cultivated on Thermanox coverslips (Nunc Thermo Scientific). Except for extracellular virions, all stages of MDV particles assembly and egress were visualized in K-cESCs (Fig. 3a), namely intranuclear A, B and C capsids (Fig. 3b), primary enveloped virions (PEV) (Fig. 3c-e), cytoplasmic C capsids (Fig. 3d) or atypical cytoplasmic mature enveloped virions (Fig. 3f), as described for MDV [9]. A striking difference from what was described earlier [9] was the abundance of primary enveloped capsids in perinuclear cisternae (Fig. 3c) or in nuclear vacuoles (Fig. 3e), as well as in cytoplasmic vacuoles (Fig. 3d), which appear similar to degenerating lamellar bodies (Fig. 3a, white circles).

**Fig. 4:**
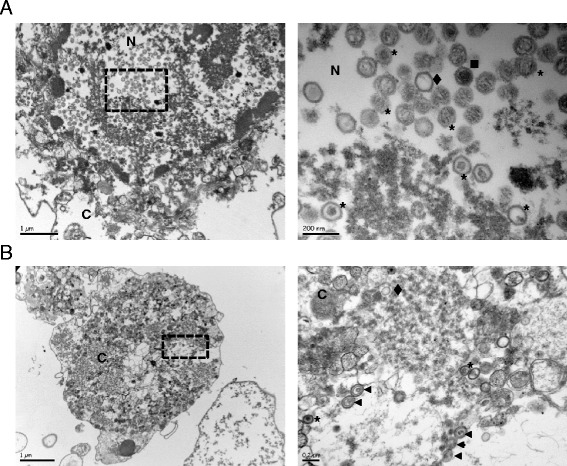
K-cESCs monolayers infected by MDV shed infected corneocytes. Pictures on the right are expansions of the areas boxed on the corresponding left-sided micrography. **a** Numerous capsids of type A (*diamond*), B (*square*) and C (*stars*) are observed in a highly modified nucleus of an infected K-cES-KP2 corneocyte. **b** Primary enveloped particles (*triangle*) and C capsids (*stars*) in infected K-cES-K1 corneocyte. Note that the corneocytes show a modified morphology with barely recognizable organelles; the nucleus could not be faithfully identified on this image. Diamond : A capsid; Square : B capsid; Stars : C capsids; Triangle : primary enveloped particles

To complete this study, floating cells from the supernatant of K-cESCs were observed by TEM (Fig. 4). We observed a large number of cells undergoing cornification, which contained numerous virions. The cornification process in those K-cESCs from the supernatant was attested by nuclear lysis, a dense network of fibres in the intracellular content, and the disappearance of organelles [17, 18]. As an example, a large amount of A, B and C capsids are shown in a considerably modified nucleus (Fig. 4a). Curiously, numerous virions with primary enveloped particle morphology were observed in the cytoplasm (Fig. 4b), although extracellular particles were not observed in these samples. From this limited study we concluded that MDV morphogenesis in keratinocytes was close to that described in CESCs. However in the keratinocytes as in CESCs, secondary enveloped particles were only rarely detected and never in electron dense large cytoplasmic inclusions as described in the transitional layer of the skin [4].

Here we show that MDV can efficiently replicate in three highly differentiated K-cESCs. These cells only produce very few secondary enveloped particles. We also showed that corneocytes shed from infected monolayers contain all types of intracellular virions. We did not find “free” enveloped virions or cytoplasmic inclusions [4]. It is noteworthy that, when using a UL47 tagged virus, we did not observe the specific enhancement of pUL47 expression which was reported for that tegument protein in the context of FFE cells [19].

We were intrigued by the frequency at which PEV were observed in both corneocytes and infected K-cESCs. The accumulation of PEV in perinuclear spaces has been associated with impaired Us3 activity [20, 21] however their presence in the cytoplasm or vacuoles is rarely reported. Whether the de-envelopment step is impaired in K-cESCs or whether this accumulation of PEV is related to the tag on UL17 remains to be ascertained. However vBAC20UL17mRFP has shown little differences with the parental vBAC20 in former studies addressing the dissemination of both viruses in primary CESCs [22] and clusters of C capsids were observed in cytoplasm of infected K-cESCs, indicating that de-envelopment and/or C capsid nuclear egress took place in K-cESCs. MDV has been reported to be impaired in secondary envelopment step in primary cells [9], which appears to be also the case in K-cESCs. This indicates that K-cESCs, although expressing most markers of differentiation in vitro [8], are unable to provide the favourable environment found by MDV in the FFE keratinocytes.

When considering the morphogenesis of MDV in the upper layers of a stratified epithelium [4], keratinocytes of the transitional layer appear to provide a specific cellular environment enabling either an efficient egress, and/or the biosynthesis of an inclusion that would protect the enveloped virions from the ultimate modifications sustained by the keratinocytes. The hypothesis of the biosynthesis of an inclusion is in good agreement with both the rare images of viral inclusions in infected FFE keratinocytes [4], and the fact that infectious “free” virus may be retrieved from feather material under rather harsh extraction conditions [3]. Whether the inclusions seen in FFE cells and, in one instance, in cultivated cells [23] are of viral origin, as described for human cytomegalovirus [24], or of cellular origin remains to be explored. In that respect, the recent identification of the differential expression of tegument proteins in FFE cells [19] is in favour of a specific activation of the expression of tegument proteins, which may facilitate the initiation of egress or inclusion formation. The shedding of infectious material “protected” from degradation in inclusions would logically account for the resistance of MDV to i) the ultimate modifications undergone by cornifying keratinocytes and ii) a rapid degradation in the environment.

The availability of K-cESCs as differentiated cell-lines that support MDV replication enables further in vitro exploration of the viral replication in 3D organized tissues to better understand the replication of MDV in the upper layers of the epidermis, expanding our knowledge on the molecular determinants associated with MDV replication and morphogenesis.

### Ethics statements

Primary chicken skin cells (CESCs) were obtained from 12 day-old embryos from LD1 Brown Leghorn chicken line [5]. This procedure was carried out in strict compliance with the French legislation for animal experiments which states that the use of embryos from oviparous species before the last third of their development (i.e. before day 14 for chicken embryos) is not submitted to regulation (Art. R.214-88).

## Abbreviations

MDV: Marek’s disease virus
FFE: Feather follicle epithelium
GaHV-2: *Gallid herpesvirus 2*
K-cESCs: Keratinocytes derived from chicken embryonic stem cells
CESCs: Primary chicken embryonic skin cells
pfu: Plaque forming unit
TEM: Transmission electron microscopy
PEV: Primary enveloped virions
